# Current News and Opinion

**Published:** 1887-02

**Authors:** 


### Current News and Opinion.

### THE IMPENETRABLE COLD ZONE IN STEAM BOILERS.

BY T. FLETCHER, F. C. S.

During my experiments on the state of things in that almost unknown space between a flame and a vessel containing water, some most extraordinary facts have come to light, which are not only of the greatest importance to steam users and boilermakers, but explain many curious points in connection with the heating of water.

It is well known that a flame does not come in contact with any ordinary vessel containing water, and that a paper label will remain on the bottom of a tin or copper kettle placed on a sharp fire, until, by drying, it gradually becomes loosened, and loses its contact with the metal, and so becomes burnt. I have myself seen labels on the bottoms of ordinary kettles and pans, the labels being quite perfect after some weeks' use over gas burners and fires. The work obtained from any source of heat by a limited surface is in direct proportion to the difference between the temperature of the vessel and that of the source of heat in absolute contact with it, and it therefore becomes a matter of serious importance to discover what the actual temperature of this cool and flameless zone is, and whether it can be removed. As is no doubt well known, my efforts to remove this, which is practically a wet blanket, from between the vessel and the fire, have been partially successful by the use of projecting studs or webs of definite proportions, and the experiments already published prove that at the ends of copper rods four diameters long, flame contact exists, at all events sufficient to char paper, and to multiply the available duty, surface for surface, six times as compared with either water tubes or ordinary boiler plates, and that the evaporating power of any properly proportioned studded or ribbed plate has no limit except the practical one of removing the steam quick enough to prevent its lifting the water bodily out of the boiler.

After proving beyond doubt that under ordinary conditions flame does not come in contact with a vessel containing water, I endeavored to get this contact and the corresponding increase in evaporating power, by directing flame against the water vessel with the assistance of a powerful blast, the result being, much to my surprise, that I found an impenetrable cold zone surrounding the vessel, absolutely impassable, not only to a powerful blow-pipe flame, urged with an air blast at 1¼ lb. per square inch pressure (the heaviest blast a glass blow-pipe will stand under ordinary conditions), but that it was equally impassable by radiant heat from a sheet of white hot platinum, held as close as possible without absolute contact In making this test the result was proved by the fact that sheets of paper pasted on the water vessel were exposed to both the direct impact of the blow-pipe flame, and also to the radiant heat from the platinum, until the water in the vessel boiled, the paper being perfectly free from charring or discoloration at the end of the test.

Another important fact came out as the result of these experiments. Not only can the maximum temperature be determined by the presence or absence of charring of known organic substances, but also the thickness or depth of the cold zone can be measured by using paper of different thickness pasted to the surface of the vessel. When the paper used is thicker than the depth of the cold zone, the surface is charred or completely burnt to an invariable depth by each source of heat; but if this charred surface is cleared off with glass paper, the under part will be found perfectly white and clean, and on again directing the flame on this clean surface it remains untouched.

This cold zone, although impassable by flame, hot air or radiant heat, is powerless to resist the carrying of heat through it by solid bodies, and while the blow-pipe flame is being directed on the paper without the slightest effect, a wire passing through the flame and touching the paper will burn it instantly and completely, although the actual temperature of the wire must of necessity be far below that of the blow-pipe flame.

The extraordinary part of the whole series of experiments seems to be the existence of a zone of cold against all surfaces of metal having water behind them, this space being, to radiant heat and flame, almost as impenetrable as the metal itself is to the water. Some heat certainly does pass, or the water would never boil; but the quantity which does make its way through is very trifling as compared with what would pass, and, in fact, what does pass, under such conditions as permit of direct flame contact with the metal.

The result of these experiments does not fit the ordinary accepted theories of radiation and absorption of heat. The fact is that the high temperature stops suddenly at a very clearly defined distance, the division line being sharply drawn. It cannot be said that the heat is absorbed at a sufficient speed to produce this cold zone, because, as a matter of fact, the heat rebounds and is dissipated to a large extent sideways, and this rebound takes place at an invariable distance from the vessel, irrespective of the angle at which the flame is driven, and depending only on the force of impact of the flame. If we could imagine the surface of the vessel covered with a layer of elastic material which is compressed by a torrent of small shot driven steadily against it, we get a mechanical representation of the actual state of things between a flame and a cold vessel, additional force of impact reducing the thickness of the elastic layer, but being powerless to annihilate it.—*Industries.*

---

### FIRST DISTRICT DENTAL SOCIETY OF THE STATE OF NEW YORK.

At a regular meeting of the above society, held Tuesday evening, January 4th, 1887, on motion of Dr. Frank Abbott, it was ordered that the secretary be instructed to correct in the next issue of the INDEPENDENT PRACTITIONER the incomplete and misleading report of the society's proceedings which appeared in the December number of that journal, page 712

The report is incomplete in that it omits to state that after the adoption of the resolutions and the appointment of a committee as provided for therein, objections were made to the legality of the action taken, on the ground that any business other than that specified in the call was not entertainable at a special meeting. It having been so decided, a motion prevailed that the resolutions be subject to ratification at the next regular meeting.

It will therefore readily be seen that the omission of so important a part from a report of the proceedings makes incorrect an otherwise correct report, and at the same time tends to mislead by making to appear as definite and final that which in reality was of no effect whatever.

The society, in regular meeting, has since refused to ratify the unlawful adoption of the resolutions, and they have been stricken from the minutes.

B. C. NASH, Secretary.

The report in question was furnished to this journal by one who was most active at the meeting, and who was therefore supposed to be unusually well qualified to give an accurate account of what was done It was accepted without question, and printed exactly as written. If there were any inaccuracies the fault was with the author of the report, and upon him should the blame rest.—EDITOR.

---

### THE DRUGGIST TO HIS BEST GIRL.

I love thee, Mary, and thou lovest me;
Our mutual flame is like the affinity
That doth exist between two single bodies;
I am Potassium to thine Oxygen.
'Tis little that the holy marriage vow
Shall shortly make us one. That unity
Is, after all, but metaphysical.
Oh, would that I, my Mary, were an acid,
A living acid; thou an alkali
Endowed with human sense, that, brought together,
We both might coalesce into one salt—
One homogeneous crystal. Oh, that thou
Wert Carbon, and myself were Hydrogen!
We would unite to form olefiant gas,
Our common coal, or naphtha. Would to heaven
That I were Phosphorus, and thou wert Lime,
And we of Lime composed a Phosphoret!
I'd be content to be Sulphuric Acid,
So that thou might be Soda; in that case
We should be Glauber's Salt. Wert thou Magnesia,
Instead, we'd form the salt, that named from Epsom.
Couldst thou Potassa be, I Aqua fortis,
Our happy union should that compound form,
Nitrate of Potash--otherwise Saltpeter;
And thus our several natures sweetly blent,
We'd live and love together until death
Should decompose the fleshy tertium quid,
Leaving our souls to all eternity
Amalgamated. Sweet, thy name is Briggs
And mine is Johnson. Wherefore should not we
Agree to form a Johnsonate of Briggs?
We will. The day, the happy day, is nigh,
When Johnson shall with beauteous Briggs combine.

—*National Druggist.*

### ST. LOUIS DENTAL SOCIETY.

The following members were elected to serve as officers for the year 1887 :

*President*—Dr. M. C. McNamara.
*Vice-President*—Dr. Henry Fisher.
*Corresponding Secretary*—Dr. W. N. Morrison.
*Recording Secretary*—Dr. John G. Harper
*Treasurer*—Dr. A. J. Prosser.

JOHN G. HARPER, *Rec. Sec'y*

816 Walnut St., St. Louis.

---

THE SCHOLARLY Dr. D. G. Brinton, editor of that paragon of medical weeklies, the Philadelphia *Medical and Surgical Reporter*, wields a pen that has a point quite as sharp as it is polished. See how neatly he punishes another editor who is always inveighling against those surgeons who get their operations reported in the newspapers in an exaggerated style. We fear, however, that the keenness of the shaft will be lost upon the man who did so much to make the General Grant case a parallel to that of Garfield :

As Dr. Geo. F. Shrady. of New York, editor of the *Medical Record*, is such a stickler for keeping the names of his fellow editors out of the newspapers, we respectfully ask his explanation of the following felicitous tribute to his skill printed in the *New York Tribune* of recent date :

"Dr. Geo. F. Shrady, of New York City, performed a noticeable surgical operation upon a young son of Mr. W. MacRussell, of Saugerties, N. Y., on Saturday last. The child's leg had been broken some time since, and it had become united so improperly that he had lost all use of it. The doctor removed a portion of the defective bone and reset the limb successfully. Various prominent surgeons witnessed the operation. The patient is doing well."

Will not our brother editor relieve us by stating that this was "without his knowledge" and "contrary to his wishes?"

---

PROF. W. D. MILLER is the first and only American who has been raised by the German government to the honorable position of professor in the Royal University of Berlin. He has discovered, described and named several micro-organisms, perhaps the most important of which are those which cause dental caries and the Miller bacillus. The latter is a comma bacillus, found in the human mouth, and which, though for a long time recognized as morphologically similar to that ascribed by Koch to cholera Asiatica, and to that by Finkler and Prior to cholera nostras, yet was so resistant of isolation by the efforts of all bacteriologists that it remained for Prof. Miller to obtain pure cultures of it only after repeated attempts.—*Journal American Medical Association.*

---

CODMAN & SHURTLEFF are manufacturing a very neat and convenient ribbon-saw carrier, devised by Dr. A. M. Dudley. Every one knows how difficult it is to make separation between back teeth, and to dress fillings in these localities without lacerating the angles of the mouth of the patient. In this carrier the saw is set at right angles to the frame, which is divided into two parts at its distal end to receive it, the arms being made of spring steel to hold it taut. Thus all danger of laceration to lips or gum is avoided, and the convenience of the operator greatly aided.

Dr. F. H. Darby, a physician of Monroe, Ohio, was summoned as a witness in a murder case. He had attended the post mortem, and when questioned, answered as to all matters of fact, but when asked for his *opinion* concerning usual results of wounds like that observed in this instance, he declined to give it unless he was guaranteed experts' fees. The presiding judge decided that he must answer the question irrespective of fees, and on the persistent refusal of Dr. Darby, he was committed to jail until he should purge himself of the contempt of court in which he was declared to stand. But Dr. Darby proved himself as firm as the judge, and after four days he was brought before the court, charges formally preferred against him, when he was admitted to bail.

The physicians of Ohio are rallying to the support of Dr. Darby, and it is proposed to carry the case to the Supreme Court of Ohio, if a favorable decision is not sooner reached. In other cases the presiding judge has sustained physicians in refusing to give a professional opinion without receiving the fees of an expert.

---

We have received from the publishers a phrenological calendar which consists of an outline of the human head, duly surveyed and laid out into town lots, each lot fully improved by a ground plan, elevation, or side view of some article or animal supposed to bear a close analogy to that portion of the brain which, in the human anatomy, forms the substratum of the lot or section. We do not know whether this is supposed to be a representation of the skull of the inventor, but notice that an ass recalcitrant forms the conspicuous ornament of one of the most eligible of the town lots. If any one should desire this kind of a calendar to hang up in his office, we should think this would be about the thing that he would like.

---

One of the most convenient things for the dentist is Pearson's Dental Appointment Book. It is of a size to be carried in the vest pocket, and yet is sufficiently large to meet all the needs of a dentist in large practice. There is no necessity for searching the whole office over for the mislaid appointment book when it is wanted, for it is always present. There is a column in which a minute of the operations of the day may be entered, preparatory to posting at night, and there are blank pages for memoranda of cases. Send fifty or seventy-five cents to R. I. Pearson & Co., Kansas City, Mo., and get it.

---

No report has yet been made by Dr. A. P. Southwick, Elbridge T. Gerry, and the Hon. Matthew Hale, constituting a committee appointed by the New York Legislature last winter to pronounce upon the most humane and effective methods for carrying out a sentence of death. The commission will probably ask for more time on account of the absence of Mr. Gerry in Europe. Dr. Southwick is said to strongly favor electricity.—*The Doctor.*

---

The Microscope for January comes to us greatly improved in appearance and enlarged in size. There are very many dentists who desire a microscopical journal, and to such we can commend *The Microscope.* $1.00 per annum. Address Detroit, Mich.

James Vick, of Rochester, has probably done more for the cultivation of a pure æsthetic taste than any man in America, for through him the love for that which is most beautiful in nature—flowers—has been made active in many a breast where it otherwise might have lain dormant during life. His Floral Guide is a work of beauty in itself, and it contains priceless information concerning the flowers and shrubs without which the most elegant residence is unfit to be a home for the family.

---

The Dental Office and Laboratory, which has so long been published by Johnson & Lund as a quarto quarterly, without cover, comes to us in its January number completely metamorphosed. It is now a handsome octavo, and is edited by Theodore F. Chupein, D. D. S., who is well and favorably known to the profession as a pithy and instructive writer. Congratulations are due the D. O. and L upon its changed and greatly improved appearance.

---

The Cincinnati Lancet and Clinic must be known to the readers of this journal, because its name is frequently seen appended to paragraphs borrowed for this department. For years we have been a reader of its pages, and always with interest and profit. Any one who desires its regular weekly visit has but to enclose $3.50 and address Dr. J. C. Culbertson, editor and publisher, Cincinnati, Ohio.

---

Caulk's Dental Annual for 1886 and 1887 is as full of statistical information as ever, and that is saying much. It is the *vade mecum* of those who desire information concerning dental schools, dental societies, and dental legislation.

---

Dr. Judson B. Andrews, Superintendent of the Buffalo Insane Asylum, has been appointed President of the section of Psychological Medicine and Nervous Diseases in the Medical Congress, in place of Dr. John P. Gray, deceased.

---

Dr. D. G. Brinton the accomplished editor of *The Medical and Surgical Reporter*, has been elected professor of American Linguistics and Archæology in the University of Pennsylvania.

---

The way to sleep, says a scientist, is to think of nothing. But Dr. Hammond asserts that this is a mistake, and says that the way to sleep is to think it is time to get up.

---

Pasteur in one year has treated by inoculation 2,490 persons believed to have been bitten by rabid animals. In ten of these cases death ensued.

Dr. Joseph G. Richardson, of Philadelphia, well known as an authority in matters of hygiene, died November 13, aged 51 years.

---

The Council of the Royal College of Surgeons of England has expelled one of its members for advertising in the secular papers.

"Charlatanism," says Oliver Wendell Holmes, "always hobbles on two crutches—the tattle of women and the certificates of clergymen."

---

The sessions of the International Medical Congress will last six days.

